# Sex differences in the efficacy and safety of SARS-CoV-2 vaccination in residents of long-term care facilities: insights from the GeroCovid Vax study

**DOI:** 10.1007/s11739-023-03283-y

**Published:** 2023-04-29

**Authors:** Caterina Trevisan, Valeria Raparelli, Alba Malara, Angela Marie Abbatecola, Marianna Noale, Annapina Palmieri, Giorgio Fedele, Anna Di Lonardo, Pasqualina Leone, Ilaria Schiavoni, Paola Stefanelli, Stefano Volpato, Raffaele Antonelli Incalzi, Graziano Onder, the GeroCovid Vax working group

**Affiliations:** 1 Department of Medical Sciences, University of Ferrara, Ferrara, Italy; 2 Department of Neurobiology, Care Sciences and Society, Aging Research Center, Karolinska Institutet and Stockholm University, Stockholm, Sweden; 3 Department of Medicine, University of Padua, Padua, Italy; 4 Department of Translational Medicine, University of Ferrara, Via Aldo Moro 8, 44124 Cona, Ferrara Italy; 5 University Center for Studies on Gender Medicine, University of Ferrara, Ferrara, Italy; 6 Scientific Committee of National Association of Third Age Residences (ANASTE) Calabria, Lamezia Terme, Catanzaro Italy; 7 Alzheimer’s Disease Day Clinic, Azienda Sanitaria Locale, Frosinone, Italy; 8 Institute of Neuroscience, National Research Council, Padua, Italy; 9 Department of Cardiovascular, Endocrine‑Metabolic Diseases and Aging, Istituto Superiore di Sanità, Rome, Italy; 10 Department of Infectious Diseases, Istituto Superiore di Sanità, Rome, Italy; 11 Campus Bio-Medico University, 9317 Rome, Italy; 12 Universita’ Cattolica del Sacro Cuore, Rome, Italy; 13 Fondazione Policlinico Gemelli IRCCS, Rome, Italy

**Keywords:** Nursing homes, SARS-CoV-2 vaccines, Safety, Efficacy, Sex differences

## Abstract

Despite the reported sex-related variations in the immune response to vaccination, whether the effects of SARS-CoV-2 vaccination differ by sex is still under debate, especially considering old vulnerable individuals, such as long-term care facilities (LTCFs) residents. This study aimed to evaluate COVID-19 infections, adverse events, and humoral response after vaccination in a sample of LTCF residents. A total of 3259 LTCF residents (71% females; mean age: 83.4 ± 9.2 years) were enrolled in the Italian-based multicenter GeroCovid Vax study. We recorded the adverse effects occurring during the 7 days after vaccine doses and COVID-19 cases over 12 months post-vaccination. In a subsample of 524 residents (69% females), pre- and post-vaccination SARS-CoV-2 trimeric S immunoglobulin G (Anti-S-IgG) were measured through chemiluminescent assays at different time points. Only 12.1% of vaccinated residents got COVID-19 during the follow-up, without any sex differences. Female residents were more likely to have local adverse effects after the first dose (13.3% vs. 10.2%, *p* = 0.018). No other sex differences in systemic adverse effects and for the following doses were recorded, as well as in anti-S-IgG titer over time. Among the factors modifying the 12-month anti-S-IgG titers, mobility limitations and depressive disorder were more likely to be associated with higher and lower levels in the antibody response, respectively; a significantly lower antibody titer was observed in males with cardiovascular diseases and in females with diabetes or cognitive disorders. The study suggests that, among LTCF residents, SARS-CoV-2 vaccination was effective regardless of sex, yet sex-specific comorbidities influenced the antibody response. Local adverse reactions were more common in females.

**Supplementary Information:**

The online version contains supplementary material available at 10.1007/s11739-023-03283-y.

## Introduction

Since the COVID-19 pandemic outbreak in 2020, more than 6 million people have died, mainly male, older, and vulnerable people with concomitant chronic non-communicable diseases [1, 2]. Undoubtedly, the advent of vaccines against Severe Acute Respiratory Coronavirus 2 (SARS-CoV-2) has strongly affected the course of the COVID-19 pandemic, mitigating the incidence and the severity of the infection in all settings. However, some knowledge gaps still exist in the immunogenicity of vaccines in some population categories, such as residents in long-term care facilities (LTCFs), undoubtedly the most vulnerable ones [3]. In this regard, an age-dependent decline in immune responses has been reported and could result in increased susceptibility to the infection and compromised vaccine immunogenicity in older, frailer individuals [4].

In addition to age, it is unclear whether sex may represent another impactful modifying factor in the efficacy and safety of SARS-CoV-2 vaccination. The presence of disparities between male and female individuals in vaccination outcomes is a well-known topic, and extensive literature has demonstrated that from childhood to adult age, females are more likely to have higher humoral and cell-mediated responses and report adverse effects than their male counterparts [5–7]. This picture may change in advanced age. Indeed, although older women showed a greater immune response to influenza vaccination, a male advantage in terms of effective response has been observed for other vaccines, such as those against pneumococcal infection and tetanus/diphtheria/pertussis [5, 7]. These differences seem to be mediated by biological (i.e., sex-related) and psycho-socio-cultural (i.e., gender-related) aspects, including genetic, hormonal, and environmental factors, that may change with aging [6, 7].

To tackle the many unknowns regarding the response to SARS-CoV-2 vaccination in female and male older people, we assessed the existence of sex differences in the efficacy (i.e., humoral response and incident SARS-CoV-2 infections) and safety (i.e., frequency and types of adverse effects) of these vaccines among older residents of LTCFs.

## Methods

### Study population

Data from this study come from the GeroCovid VAX study, an ongoing multicenter project promoted by the Italian Society of Gerontology and Geriatrics (SIGG, Florence, Italy) and the Istituto Superiore di Sanità (ISS, Rome, Italy) and sponsored by the Italian Medicines Agency (AIFA). The detailed study protocol has already been published [8, 9]. Briefly, the study aimed to evaluate the efficacy and safety of vaccination against SARS-CoV-2 in older residents of LTCFs in Italy.

The vaccination campaign in Italian LTCFs began at the end of December 2020 from the LTCFs and used mainly mRNA vaccines (Moderna mRNA-1273 or Cominarty BNT162b2). According to national guidelines, residents who got SARS-CoV-2 infection in the prior 6 months received only one vaccine dose, while the others were administered a second dose after 4 weeks from the first one [10]. A booster dose was offered between October and November 2021 (around 8 months after the first dose).

Participants (*n* = 3268) underwent clinical monitoring of possible vaccine adverse effects, incident COVID-19, Emergency Department (ED) accesses, unplanned hospitalizations, and mortality after 7 days from the first, second, and booster doses and after 2, 6, and 12 months from the first dose administration.

From the overall population, for this study, we excluded 6 individuals for whom information on sex was not recorded and 3 participants who had neither data on adverse effects nor the possible occurrence of COVID-19 over the 12-month follow-up, obtaining a final analytical sample of 3259 residents.

In a representative subgroup of participants (*n* = 524), we could also perform serological monitoring before the vaccination and after 2, 6, and 12 months from the first vaccine dose administration.

The study protocol received approval from the Italian National Ethical Committee (permission number 264/2021; January 26, 2021) and the Ethical committee at each participating center.

### Data collection

Data collection was performed by physicians or researchers skilled in the geriatric field after appropriate training in an electronic platform developed by Bluecompanion Ltd (London, UK). For each participant, we collected sociodemographic information (age, sex) and clinical and functional status data. As a proxy of functional status, we considered mobility level, categorized as high (moving independently with or without walking aids) vs low (moving with a wheelchair or bedridden). The presence of chronic diseases was ascertained by physicians based on the medical records at the participant’s LTCF and the list of ongoing medications. For this study, the following conditions were considered: diabetes mellitus, osteoarticular diseases (including osteoarthritis and osteoporosis), hypertension, cardiovascular diseases (CVD, including atrial fibrillation ischemic, arrhythmic or valvular heart diseases, and heart failure), chronic respiratory diseases, obesity, depressive disorders, anxiety, Parkinson’s disease or parkinsonism, thyroid disorders, epilepsy, hyperuricemia/gout, urologic disorders, gynecologic diseases, dermatologic diseases, chronic liver diseases, biliary disorders, eye/ear/nose/throat disorders, previous stroke, chronic kidney failure, cancer, immune system disorder, and inflammatory bowel diseases. The total sum of chronic diseases was obtained from the count of the above-listed conditions. Moreover, anamnestic information on cognitive disorders, including either a diagnosis of dementia or the presence of cognitive impairment from the cognitive evaluation at the LTCF, was also collected. Finally, from the list of the drugs chronically used by the participants, we derived data on ongoing therapies potentially influencing immune function (e.g. steroids, antiinflammatory or immunomodulatory drugs).

#### SARS-CoV-2 infection history and vaccination data

Information on previous SARS-CoV-2 infection (confirmed by Real-Time PCR testing) was collected for each participant at baseline. Concerning SARS-CoV-2 vaccination, we recorded information on the date, type, and number of vaccine doses received.

#### Vaccine adverse effects

Information on possible adverse effects was collected during the 7 days after the first, second, and booster vaccine doses. In particular, according to the current literature, we systematically assessed the occurrence of side effects with an ad hoc questionnaire. Among the local side effects, we considered pain and swelling, itching, or redness at the injection site; among the systemic adverse effects, we considered: fever, low-grade fever, muscle weakness, muscles and joints pain, headache, swollen lymph nodes, chills, difficulty breathing, insomnia, sneezing, fast heart rate, cough, anorexia, nausea or vomiting, delirium, diarrhea, increased blood pressure, weakness, cutaneous rash, confusion, and dizziness.

#### Incident SARS-CoV-2 infection

Over 12 months from the first vaccine dose, incident SARS-CoV-2 infections were recorded. In particular, for each participant, we collected information on the date of the positive nasopharyngeal swab for SARS-CoV-2 determination and the severity of the disease according to the World Health Organization classification. For the purpose of this study, we categorized disease severity as: asymptomatic, mild disease with no oxygen requirements, mild or severe disease with oxygen or organ support needs, and death.

#### Biochemical data

In a random sample of residents, the humoral immune response was evaluated at baseline (i.e., before the vaccination, T0) and after 2 (T1), 6 (T2), and 12 (T3—post-booster dose) months from the first vaccine dose. Blood samples were prepared and stored according to a standardized procedure. Fasting blood samples were collected in the morning in Serum Separator Tubes (B.D. Diagnostic Systems, Franklin Lakes, NJ, USA) and centrifuged at room temperature at 1600 rpm for 10 min. Aliquots were transferred to 2 ml polypropylene screw cap cryotubes (Nunc™, Thermofisher Scientific, Waltham, MA USA). Frozen sera were stored at − 80 °C and analyzed at the ISS laboratory. SARS-CoV-2 IgG (anti-S IgG) was measured through Liaison® SARS-CoV-2 Trimeric S IgG chemiluminescent assay (DiaSorin, Italy), using the trimeric S antigen stabilized in its native form and developed for high throughput. The LIAISON® XL fully automated chemiluminescence analyzer automatically computes SARS-CoV-2 trimeric S IgG antibody titers, measured in binding antibody units (BAU/ml). The upper measurable limit of the assay is 2080 BAU/ml. In line with the manufacturer’s recommendations, antibody titers equal or greater than 33.8 BAU/ml were defined as positive. Samples whose antibody levels overcame the assay’s upper limit were diluted 1:20 and re-analyzed.

### Statistical analysis

The characteristics of the male and female participants were expressed as mean (standard deviation, SD) for the continuous variables and as count (%) for the categorical variables. The comparison between male and female characteristics and adverse effects frequency was performed through the Student *t* test and Chi-squared test, as appropriate.

The association between sex and the risk of reporting SARS-CoV-2 infection over the 12-month follow-up was evaluated through Cox regression after verifying the proportional hazard assumption. The model was first adjusted for age (Model 1) and then also for other possible confounders, i.e., ethnicity, mobility level, previous SARS-CoV-2 infection, number of vaccine doses received, cognitive disorders, and number of chronic diseases (Model 2).

The evaluation of anti-SARS-CoV-2 IgG levels in males and females who participated in the serological monitoring was performed after the log-transformation of antibody titers due to their non-normal distribution. The presence of sex differences in the extent of antibody response at T1, T2, and T3, compared to T0, was evaluated by testing the interaction between sex and time in linear mixed models (with a random intercept), adjusted, first, by age, ethnicity, and previous SARS-CoV-2 infection (time-varying variable) (Model 1), and, second, also for the number of vaccine doses received, mobility level, cognitive disorders, the number of chronic diseases, and the use of drugs potentially influencing the immune system (Model 2).

For both the above-described analyses, variables included in Model 2 were selected based on the evidence in the current literature of possible confounders in the studied association. In particular, previous studies demonstrated that some sociodemographic (e.g. age and ethnicity), health- and frailty-related (clinical complexity measured with the number of chronic diseases, functional status, and mobility level), and pharmacological (e.g. use of immunomodulatory drugs) factors might influence the effectiveness of vaccines (in terms of prevention from new infections and extent of the humoral response) and the occurrence of side effects [11].

Sex-stratified linear mixed models (with random intercept) were run to evaluate the possible modifying role of mobility level (as a proxy of functional status) and the most prevalent clinical conditions in our population (at least 20% prevalence) that could affect antibody kinetics, i.e., CVD, chronic respiratory diseases, diabetes mellitus, osteoarticular diseases, anxiety, depressive and cognitive disorders. For this purpose, analyses included the interactions between the above-listed variables and time and were adjusted for age, ethnic origin, previous SARS-CoV-2 infection (time-varying variable), and the number of vaccine doses received.

Statistical analyses were performed using R statistical software [12].

## Results

### Characteristics of the sample

Of the 3259 LCTF residents, 2318 (71.1%) were females, and 97.8% were Caucasic. The entire sample’s mean age was 83.4 (SD 9.2) years, and one-third (33.3%) had low mobility. The characteristics of male and female participants are shown in Table 1. Compared with males, female residents were significantly older and less likely to move independently or with walking aids. The burden of morbidities was similar regardless of sex (mean number of chronic diseases = 5.34). Overall, the most prevalent conditions were hypertension (70.7%), cognitive disorders (63.9%), CVD (55.9%), osteoarticular diseases (51.9%), and depressive disorders (51.5%). Female residents showed a higher frequency of hypertension, osteoarticular diseases, and cognitive and depressive disorders. Instead, males were more likely to have cerebrovascular and respiratory diseases, diabetes, chronic liver, renal, and urologic diseases, and Parkinson’s disease or parkinsonism. Regarding the type of SARS-CoV-2 vaccine, the majority (87.4%) of participants’ first doses were Cominarty BNT162b2, and, over the study period, most of them received two (41.5%) or three doses (51%), with no differences by sex. Females were more likely to have previously been infected by SARS-CoV-2 than men (32.3% vs. 27.9%, *p* = 0.02).

**Table 1:** Baseline characteristics of the male and female study participants

	Men (*n* = 941)	Women (*n* = 2318)	*p *value
Age (years)	79.53 (9.69)	84.91 (8.44)	< 0.001
Mobility level			0.002
Walks independently or with aids	593 (63.0)	1305 (56.3)	
Moves with a wheelchair or bedridden	276 (29.3)	808 (34.9)	
Chronic diseases			
Hypertension	628 (66.7)	1677 (72.3)	0.002
Cardiovascular diseases	511 (54.3)	1311 (56.6)	0.256
Cerebrovascular diseases	165 (19.9)	311 (15.6)	0.007
Chronic respiratory diseases	224 (23.8)	386 (16.7)	< 0.001
Diabetes mellitus	243 (25.8)	463 (20.0)	< 0.001
Obesity	55 (5.8)	219 (9.4)	0.001
Chronic liver disease	78 (9.1)	146 (6.9)	0.048
Immune system disorder	32 (3.7)	96 (4.5)	0.379
Inflammatory bowel disease	21 (2.4)	52 (2.4)	1.00
Cancer	131 (15.2)	267 (12.6)	0.061
Osteoarticular diseases	349 (37.1)	1341 (57.9)	< 0.001
Chronic renal failure	138 (16.0)	231 (10.9)	< 0.001
Urologic diseases	243 (25.8)	13 (0.6)	< 0.001
Cognitive disorders	538 (57.2)	1546 (66.7)	< 0.001
Parkinson’s disease or parkinsonism	114 (12.1)	192 (8.3)	0.001
Depressive disorders	436 (46.3)	1241 (53.5)	< 0.001
Anxiety disorders	188 (20.0)	516 (22.3)	0.165
N. chronic diseases	5.34 (2.55)	5.34 (2.45)	0.995
COVID-19 vaccine type (first doses)			0.226
Moderna	58 (6.2)	159 (6.9)	
Comirnaty	882 (93.7)	2159 (93.1)	
AstraZeneca	1 (0.1)	0 (0.0)	
Number of vaccine doses received			0.267
1	76 (8.1)	169 (7.3)	
2	406 (43.1)	947 (40.9)	
3	459 (48.8)	1202 (51.9)	
Previous COVID-19	234 (27.9)	637 (32.3)	0.023

*n* = 77 participants had missing information on mobility level

### Local and systemic adverse effects

**Fig. 1:**
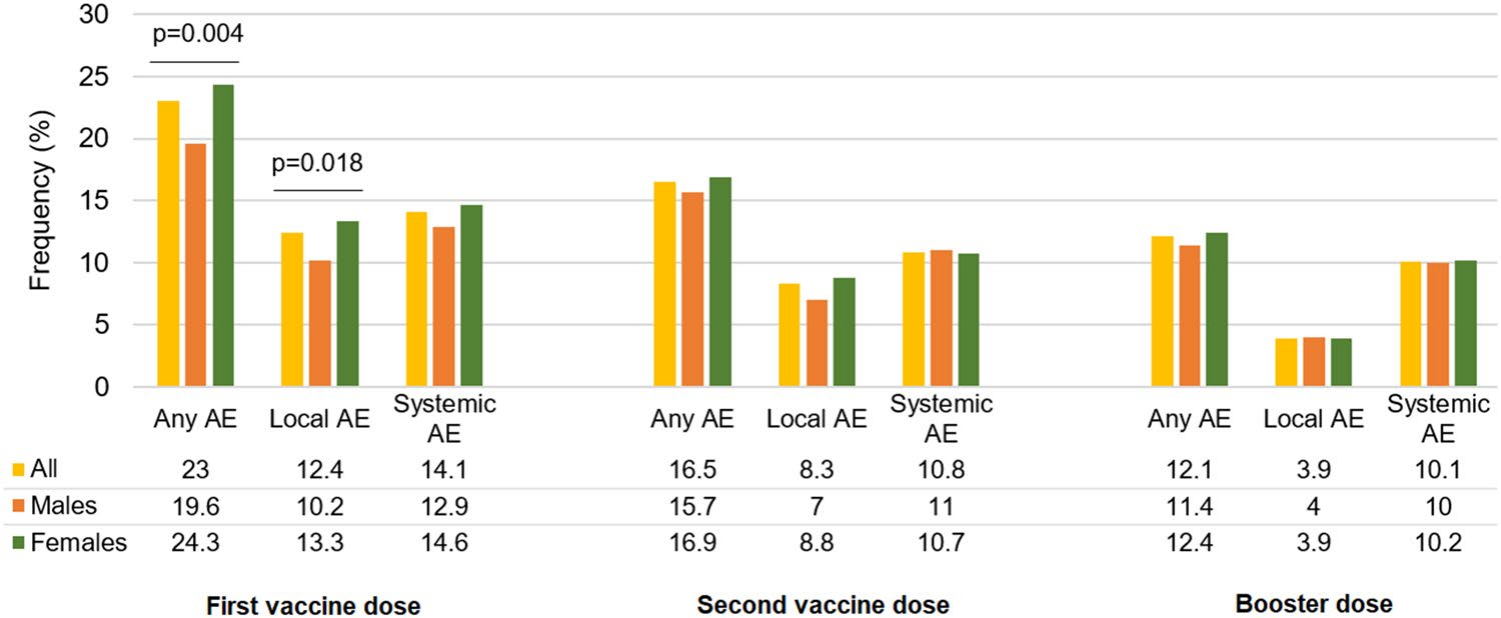
Frequency of adverse effects in the 7 days after the first, second or booster vaccine dose in male and female participants. *A.E.* adverse effect. Local adverse effects include pain and swelling, itching, or redness at the injection site. Systemic adverse effects include fever, muscle weakness, low-grade fever, muscle and joints pain, headache, swollen lymph nodes, chills, difficulty breathing, insomnia, sneezing, fast heart rate, cough, anorexia, nausea or vomiting, delirium, diarrhea, increased blood pressure, weakness, cutaneous rash, confusion, or dizziness

Figure 1 shows the frequency of local and systemic adverse effects of SARS-CoV-2 vaccines in males and females. Of the sample, 23% reported at least one adverse effect after the first vaccine dose, 16.5% after the second, and 12.1% after the third dose. The most frequent local adverse effects observed after the first dose were pain and swelling (9%), redness (4.1%), and itching at the injection site (2.5%). Among the systemic adverse effects, we mainly recorded muscle and joint pain (3.8%), fever (2.7%), and weakness (2.5%). Females were significantly more likely than males to report local adverse effects after the first dose, especially redness at the injection site; no other relevant differences were found between the sexes (Online Resource 2).

### Incident SARS-CoV-2 infections

Over a median follow-up of 365 days (IQR: 183–365), 395 individuals (123 M, 272 F) were affected by COVID-19 at least once. The cumulative incidence of COVID-19 was 13.1% in male vs 11.7% in female participants (*p* = 0.311). Among the 395 individuals who got SARS-CoV-2 infection, most were asymptomatic (88.6% M vs 87.9% F), while only a minority had mild disease with no oxygen requirements (9.8% M vs 11.8% F) or died (1.6% M vs 0.4% F), with no differences by sex (*p* = 0.354, Online Resource 3). The univariate and multivariable Cox regressions (Online Resource 4) corroborated these results since no significant associations emerged between sex and incident SARS-CoV-2 infection.

### Antibody response to SARS-CoV-2 vaccines

**Fig. 2:**
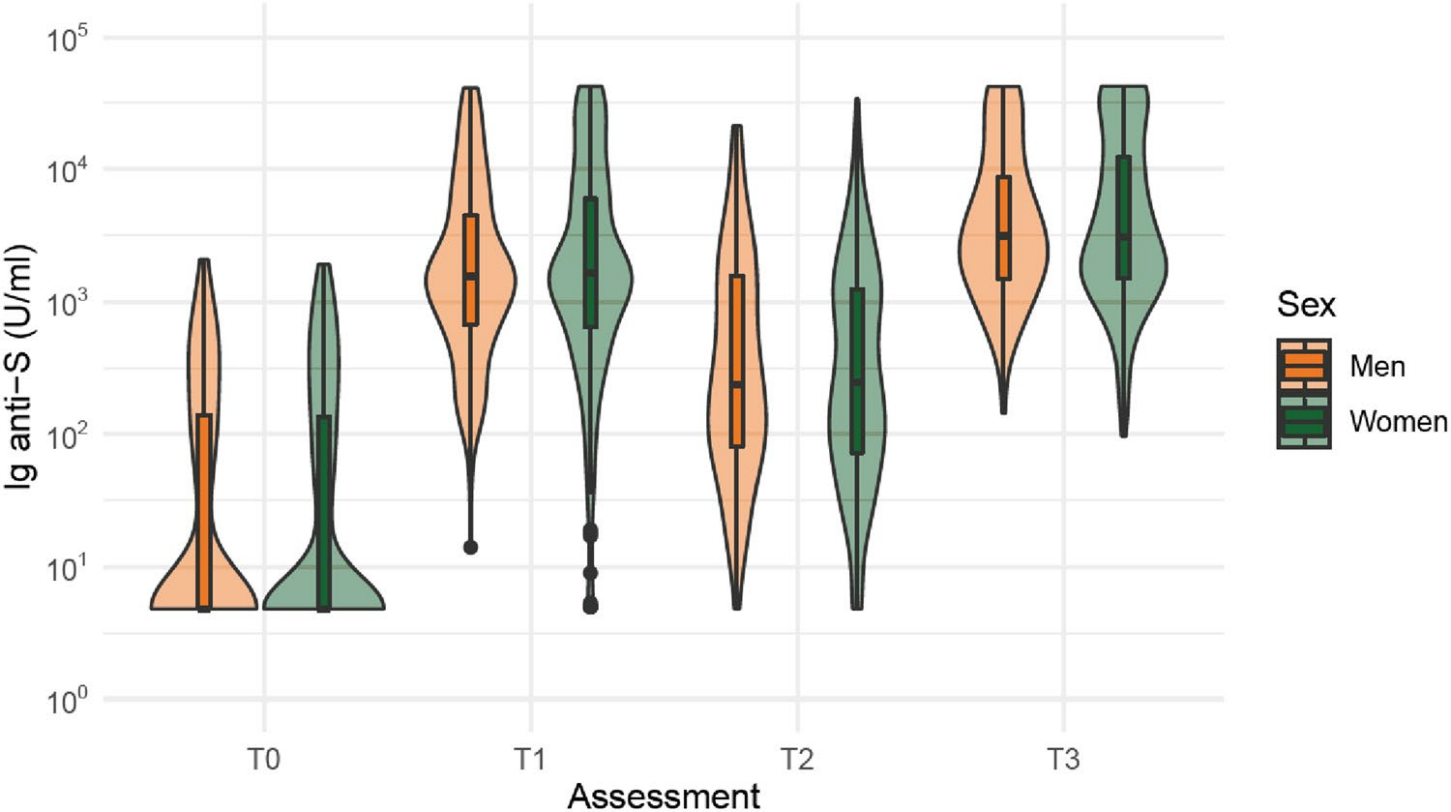
Violin plots on the anti-S IgG levels in men and women before vaccination and after 2, 6, and 12 months from the first vaccine dose administration. *T1* 2-month assessment, *T2* 6-month assessment, *T3* 12-month assessment

The characteristics of the male and female residents who underwent the serological monitoring are reported in Online Resource 5, while their anti-S IgG levels over time are shown in Fig. 2. As confirmed from the linear mixed models (Table 2), no substantial sex-based differences were observed over time in the IgG levels, regardless of the number of vaccine doses and previous COVID-19. When investigating the factors associated with antibody response to the vaccine (Table 3), we found that in both sexes, having lower mobility level was associated with increased antibody levels at 12 months, while those with depressive disorders tended to have lower antibody titers. Only among females, lower antibody concentrations at each assessment emerged in those with diabetes and at 12 months in those with cognitive disorders. Instead, males with CVD had decreased antibody titers at 12 months (Table 3).

**Table 2:** Multivariable linear mixed model for the association between sex and log10-transformed anti-S antibodies in older residents before vs after 2, 6, and 12 months from the vaccination

	*β*-Coefficient (95% confidence interval) *p *value	
	Model 1	Model 2
Sex (female vs male)	0.062 (− 0.097, 0.222) *p* = 0.441	0.080 (− 0.071, 0.231) *p* = 0.299
Sex * time		
T1 * sex (female vs male)	0.022 (− 0.130, 0.174) *p* = 0.778	0.030 (− 0.122, 0.182) *p* = 0.701
T2 * sex (female vs male)	− 0.014 (− 0.176, 0.148) *p* = 0.863	− 0.021 (− 0.184, 0.142) *p* = 0.799
T3 * sex (female vs male)	0.083 (− 0.098, 0.264) *p* = 0.368	− 0.015 (− 0.195, 0.165) *p* = 0.867

Model 1 includes age, sex, ethnic origin, COVID-19 infection in the prior period (time-varying variable), and time. Model 2 also includes the number of vaccine doses received, mobility level, cognitive disorders, the use of drugs potentially influencing immune response, and the number of chronic diseases

*T1* 2-month assessment, *T2* 6-month assessment, *T3* 12-month assessment

**Table 3:** Linear mixed model for the changes in log10-transformed anti-S antibodies as a function of sociodemographic, functional, and clinical variables in older residents over 12 months from the vaccination

	*β*-Coefficient (95% confidence interval) *p* value		
	T1 (2 months)	T2 (6 months)	T3 (12 months)
**Male residents**			
Low mobility level	− 0.029 (− 0.31, 0.251)	0.016 (− 0.290, 0.322)	0.448 (0.090, 0.807)*
Cardiovascular diseases	− 0.045 (− 0.311, 0.222)	0.011 (− 0.279, 0.301)	− 0.411 (− 0.731, − 0.091)*
Respiratory diseases	0.222 (− 0.080, 0.524)	0.080 (− 0.261, 0.421)	0.114 (− 0.266, 0.493)
Diabetes mellitus	− 0.005 (− 0.300, 0.291)	0.048 (− 0.273, 0.368)	0.040 (− 0.306, 0.386)
Osteoarticular diseases	0.161 (− 0.135, 0.456)	0.093 (− 0.244, 0.430)	0.207 (− 0.165, 0.580)
Anxiety disorders	0.097 (− 0.191, 0.385)	− 0.089 (− 0.388, 0.210)	0.137 (− 0.196, 0.471)
Depressive disorders	− 0.330 (− 0.589, − 0.071)*	− 0.172 (− 0.448, 0.103)	− 0.177 (− 0.475, 0.122)
Cognitive disorders	− 0.025 (− 0.282, 0.233)	− 0.044 (− 0.320, 0.231)	0.068 (− 0.237, 0.374)
**Female residents**			
Low mobility level	− 0.034 (− 0.213, 0.144)	− 0.043 (− 0.234, 0.148)	0.274 (0.053, 0.495)*
Cardiovascular diseases	0.136 (− 0.037, 0.310)	− 0.009 (− 0.195, 0.176)	0.068 (− 0.145, 0.281)
Respiratory diseases	− 0.017 (− 0.242, 0.208)	− 0.046 (− 0.287, 0.195)	− 0.110 (− 0.389, 0.169)
Diabetes mellitus	− 0.301 (− 0.509, − 0.094)**	− 0.325 (− 0.543, − 0.107)**	− 0.290 (− 0.546, − 0.033)*
Osteoarticular diseases	0.071 (− 0.110, 0.252)	− 0.047 (− 0.239, 0.145)	0.085 (− 0.135, 0.306)
Anxiety disorders	0.011 (− 0.200, 0.222)	− 0.006 (− 0.227, 0.216)	− 0.040 (− 0.285, 0.205)
Depressive disorders	− 0.015 (− 0.184, 0.153)	− 0.092 (− 0.273, 0.088)	− 0.235 (− 0.441, − 0.028)*
Cognitive disorders	0.037 (− 0.152, 0.226)	0.049 (− 0.152, 0.249)	− 0.272 (− 0.501, − 0.043)*

The model is adjusted also for age, ethnic origin, COVID-19 infection in the prior period (time-varying variable), the use of drugs potentially influencing immune response, and the number of vaccine doses received

*T1* 2-month assessment, *T2* 6-month assessment, *T3* 12-month assessment

*p < 0.05; **p < 0.01

## Discussion

Among older and multimorbid residents of LTCF, SARS-CoV-2 vaccination had similar efficacy in males and females. Yet, sex-related differences emerged in the safety profile and the factors associated with the antibody response to the vaccination. In fact, the frequency of local adverse effects after the first vaccine dose was higher in female participants than in males. Moreover, although LTCF residents showed similar antibody kinetics over 12 months, some of the chronic conditions associated with humoral response differed by sex.

Although vaccination had an impact on the restraint of the SARS-CoV-2 infection, the duration of protection, the optimal timing of the vaccination, and the identification of the factors associated with an effective antibody response have still to be fully clarified. Understanding clinical factors that impact the strength and duration of the immune response to vaccines represents a goal of preventive strategies, particularly among those mostly affected by the fatal consequences of the disease, such as older people living in LTCFs. As broadly reported, people in advanced age have been the category of individuals the most hit by the COVID-19 pandemic in terms of incident cases and fatality rate, so they have been the first target of the vaccination campaign [10].

While some studies have reported the effect of isolated or few conditions on the immune response after the vaccination among middle-aged individuals [13–15], data on older people are scarce. The lack of information on the most vulnerable people also concerns the adverse effects of SARS-CoV-2 vaccines and their differences by sex. Overall, although several community-based studies found that younger and female individuals were more likely to report mild adverse effects, others recorded more serious reactions among older people and in men, or no sex-related differences [16–18]. In the present work, it was evidenced that 23% of the enrolled people had adverse effects after the first vaccine dose, with women being more likely than men to present local reactions. The frequency of adverse effects decreased after the second and subsequent booster doses and did not show any other substantial difference by sex.

Our findings align with and reinforce the higher prevalence of adverse effect reactions after vaccination in women. It is well known that older females are generally more likely to report adverse reactions than males in response to different vaccines, regardless of the reaction type (local vs systemic) [7]. As emerged from information of the Vaccine Adverse Event Reporting System (VAERS) in the U.S. for seasonal influenza, the frequency of local erythema and induration, markers of local inflammation, at the site of injection was higher in aged females. Whether differences in adverse reactions reflect a sex-based reporting bias or a real sex difference in inflammatory response has not been clearly understood. Furthermore, the increased incidence of local reactions, including tenderness and pain at the injection site after the first dose of SARS-CoV-2 vaccination, has been reported in both retrospective and prospective studies [19, 20].

In our sample, we did not observe any difference in antibody titer changes over time in males and females, nor the risk of SARS-CoV-2 breakthrough infection after vaccination. This issue contrasts with the results of studies including individuals with a younger age and a better health status than our participants [21]. Instead, one previous work involving also older adults showed that the slight sex disparities in antibody titers after the primary vaccine cycle disappeared after the booster dose administration [22].

Thanks to the large set of variables collected for each participant, the GeroCovid Vax study allowed us to explore this issue and answer whether functional and clinical factors can affect the magnitude of immune response to SARS-CoV-2 vaccination. Moreover, we could investigate if the pattern of these factors varies by sex, as actually demonstrated by our findings. The sex-stratified analyses on the factors associated with a lower and heterogenous antibody response after vaccination revealed some intriguing findings. Among older male residents, those with CVD were more likely to have a lower antibodies response compared to CVD-free participants. As far as CVD are concerned, previous studies provide results similar to ours since antibody titers were found to be decreased in individuals with chronic heart disease or those with more severe congenital cardiopathies [23, 24]. Conversely, another work did not see any apparent difference in response to influenza vaccination between patients with coronary artery diseases and controls [25]. In our study, the negative impact of CVD on humoral immune response was observed only among men. The reasons behind a poor humoral response to the SARS-CoV-2 vaccine are still unclear. In a small retrospective analysis, lower antibodies titer was documented among subjects with CVD in response to the BNT162b2 mRNA SARS-CoV-2 vaccine after the first and second doses independently by sex, and the authors suggested an interaction with the cardiovascular medications of these patients [26]. Nevertheless, our findings might be influenced by the higher prevalence of CVD in the male population.

Among female residents, we found a negative influence on the vaccine humoral response from diabetes and cognitive disorders. The greater impact of diabetes on vaccine immunogenicity in female participants has already emerged from a previous study in the same population [27]. In that work, while admitting a possible residual confounding due to the generally worse health status in older male residents compared to females, we underlined the need for further studies evaluating whether biological factors, e.g. sex hormones variations, drive such differences. Regarding cognitive disorders, our findings align with a previous study observing lower antibody levels after influenza vaccination in people with dementia [28]. For instance, Ward et al. found that individuals affected by neurological diseases were less likely to present adequate antibody production against SARS-CoV-2 after 21 days of vaccination completion [29]. One of the explanations for this result concerns the high co-existence of poor nutritional status and depressive symptoms in people with neurocognitive disorders, which have both been associated with a weaker antibody production [30–32]. Moreover, the changes in the immune system occurring with aging and exacerbated in people with dementia could further attenuate the adaptive immunity and, therefore, antibody response [33].

Finally, in both sexes, depressive disorders were associated with lower antibody titers, in accordance with a large amount of literature that underlined a link between depressive mood and dysfunctions of innate and acquired immune responses [30]. For instance, a weaker cellular-mediated response to the herpes zoster vaccine was observed in older individuals suffering from major depression [34]. This finding corroborates previous studies and underlines a link between psychological stress and immune response to influenza vaccination, especially in older people [35, 36]. Vaccine-induced immunity is a complex response involving humoral and cellular responses that simultaneously orchestrate the protection against the infection. Despite only a few works evaluating possible sex-related differences, the detrimental effect of states of psychological distress on vaccination response in the current literature seems similar in men and women [37]. Conversely, the results about the increased antibody levels at 12 months among those with a low mobility level are not of univocal interpretation. Indeed, results on the influence of functional status on the immune response to vaccines are contrasting, with some studies reporting weaker responses in people with disabilities and others showing no significant associations [38, 39]. Furthermore, our results could be driven by survival bias, which led to selecting the “healthiest” ones among vulnerable individuals at the 12-month follow-up. Therefore, this issue needs to be verified in other works that better explore the influence that inflammaging-related changes [40–42] can play on the efficacy and safety of common vaccines. In this regard, inflammation should be seen as an adaptive phenomenon that tries to limit pro-inflammatory processes occurring with aging through continuously stimulating anti-inflammatory responses. Therefore, maintaining an optimal balance between inflammation and anti-inflammation represents the crucial point in determining the effectiveness of immune systems against the onset of acute or chronic diseases in advanced age [43]. Whether the intersection between the ability to keep an immune balance and sex may further impact the immune response to vaccination in older individuals is still a matter of debate [44, 45].

The strengths of the present analyses are the sample size of older male and female residents and the large set of collected variables. However, our findings should be carefully interpreted in light of several limitations. First, evaluating the factors associated with a higher/lower efficacy and safety of the vaccination in LTCF residents was one of the study’s aims but not the primary objective. Therefore, despite including a representative number of older male and female residents, the study sample size was not computed based on pre-planned sex-stratified analyses. Second, we described the antibody titers at specified time points and limited the observation to 12 months after the first vaccine dose; therefore, we cannot exclude that the kinetics and magnitude of antibody response might have varied at different time points between older females and males. Third, we did not consider data on the severity of the diseases that might have interfered with an effective immune response. Finally, the higher incidence of local adverse events among female participants might result from a reporting bias, as it was previously documented that female subjects are more likely to report adverse drug reactions [46].

## Conclusions

SARS-CoV-2 vaccination seems to have similar efficacy in older male and female LTCF residents. However, some sex-specific differences emerged in the occurrence of local adverse effects of the vaccination and in patterns of factors that influence antibody levels including functional and medical characteristics. Overall, this supports the need for a sex- and gender-specific approach when conducting research on vaccination response, also in older age.

## Supplementary Information

Below is the link to the electronic supplementary material.

Supplementary file 1 (DOCX 31 KB)

## Data Availability

The data that support the findings of this study are available from the Principal Investigator of the GeroCovid Vax initiative (Prof Graziano Onder; graziano.onder@unicatt.it) upon reasonable request.

## References

[1] Dong E, Du H, Gardner L (2020). An interactive web-based dashboard to track COVID-19 in real time. Lancet Infect Dis.

[2] The sex, gender and COVID-19 project | global health 50/50. https://globalhealth5050.org/the-sex-gender-and-covid-19-project/. Accessed 29 May 2022

[3] Antonelli Incalzi R, Trevisan C, Del Signore S (2021). Are vaccines against COVID-19 tailored to the most vulnerable people?. Vaccine.

[4] Pereira B, Xu XN, Akbar AN (2020). Targeting inflammation and immunosenescence to improve vaccine responses in the elderly. Front Immunol.

[5] Klein SL, Flanagan KL (2016). Sex differences in immune responses. Nat Rev Immunol.

[6] Ruggieri A, Anticoli S, D’ambrosio A (2016). The influence of sex and gender on immunity, infection and vaccination. Ann Ist Super Sanita.

[7] Fink AL, Klein SL (2015). Sex and gender impact immune responses to vaccines among the elderly. Physiology (Bethesda).

[8] Abbatecola AM, Incalzi RA, Malara A (2022). Monitoring COVID-19 vaccine use in Italian long term care centers: the GeroCovid VAX study. Vaccine.

[9] Abbatecola AM, Antonelli Incalzi R, Malara A (2022). Disentangling the impact of COVID-19 infection on clinical outcomes and preventive strategies in older persons: an Italian perspective. J Gerontol Geriatr.

[10] Ministero della Salute (2020) Raccomandazioni per l’organizzazione della campagna vaccinale contro SARS-CoV-2/COVID-19 e procedure di vaccinazione

[11] Zimmermann P, Curtis N (2019). Factors that influence the immune response to vaccination. Clin Microbiol Rev.

[12] R Development Core Team (2008) R: a language and environment for statistical computing. R Foundation for Statistical Computing, Vienna. ISBN 3-900051-07-0. http://www.R-project.org

[13] Herishanu Y, Avivi I, Aharon A (2021). Efficacy of the BNT162b2 mRNA COVID-19 vaccine in patients with chronic lymphocytic leukemia. Blood.

[14] Dispinseri S, Lampasona V, Secchi M (2021). Robust neutralizing antibodies to SARS-CoV-2 develop and persist in subjects with diabetes and COVID-19 pneumonia. J Clin Endocrinol Metab.

[15] Carr EJ, Wu M, Harvey R (2021). Neutralising antibodies after COVID-19 vaccination in UK haemodialysis patients. Lancet.

[16] Urakawa R, Isomura ET, Matsunaga K (2022). Impact of age, sex and medical history on adverse reactions to the first and second dose of BNT162b2 mRNA COVID-19 vaccine in Japan: a cross-sectional study. BMC Infect Dis.

[17] Montano D (2022). Frequency and associations of adverse reactions of COVID-19 vaccines reported to pharmacovigilance systems in the European Union and the United States. Front Public Health.

[18] Beatty AL, Peyser ND, Butcher XE (2021). Analysis of COVID-19 vaccine type and adverse effects following vaccination. JAMA Netw Open.

[19] Menni C, Klaser K, May A (2021). Vaccine side-effects and SARS-CoV-2 infection after vaccination in users of the COVID symptom study app in the UK: a prospective observational study. Lancet Infect Dis.

[20] Hoffmann F, Allers K (2017). Variations over time in the effects of age and sex on hospitalization rates before and after admission to a nursing home: a German cohort study. Maturitas.

[21] Klein SL, Pekosz A, Park HS (2020). Sex, age, and hospitalization drive antibody responses in a COVID-19 convalescent plasma donor population. J Clin Invest.

[22] Shapiro JR, Sitaras I, Park HS (2022). Association of frailty, age, and biological sex with severe acute respiratory syndrome coronavirus 2 messenger RNA vaccine-induced immunity in older adults. Clin Infect Dis.

[23] Zhao M, Slotkin R, Sheth AH (2022). Clinical variables correlate with serum neutralizing antibody titers after COVID-19 mRNA vaccination in an adult, US-based population. medRxiv Prepr Serv Health Sci.

[24] Fusco F, Scognamiglio G, Merola A (2021). COVID-19 vaccination in adults with congenital heart disease: real-world data from an Italian tertiary centre. Int J Cardiol Congenit Heart Dis.

[25] Keshtkar-Jahromi M, Vakili H, Rahnavardi M (2009). Antibody response to influenza immunization in coronary artery disease patients: a controlled trial. Vaccine.

[26] Naruse H, Ito H, Izawa H (2021). Immunogenicity of BNT162b2 mRNA COVID-19 vaccine in patients with cardiovascular disease. J Clin Med.

[27] Virgilio E, Trevisan C, Abbatecola A (2022). Diabetes affects antibody response to SARS-CoV-2 vaccination in older residents of long-term care facilities: data from the GeroCovid Vax study. Diabetes Care.

[28] Bellei NCJ, Carraro E, Castelo A, Granato CFH (2006). Risk factors for poor immune response to influenza vaccination in elderly people. Braz J Infect Dis.

[29] Ward H, Whitaker M, Flower B (2022). Population antibody responses following COVID-19 vaccination in 212,102 individuals. Nat Commun.

[30] Beurel E, Toups M, Nemeroff CB (2020). The bidirectional relationship of depression and inflammation: double trouble. Neuron.

[31] Bourke CD, Berkley JA, Prendergast AJ (2016). Immune dysfunction as a cause and consequence of malnutrition. Trends Immunol.

[32] Lin T-Y, Hung N-K, Hung S-C (2022). Association of malnutrition with SARS-CoV-2 vaccine response in patients undergoing hemodialysis. Clin Nutr.

[33] Lutshumba J, Nikolajczyk BS, Bachstetter AD (2021). Dysregulation of systemic immunity in aging and dementia. Front Cell Neurosci.

[34] Irwin MR, Levin MJ, Laudenslager ML (2013). Varicella zoster virus-specific immune responses to a herpes zoster vaccine in elderly recipients with major depression and the impact of antidepressant medications. Clin Infect Dis.

[35] Pedersen AF, Zachariae R, Bovbjerg DH (2009). Psychological stress and antibody response to influenza vaccination: a meta-analysis. Brain Behav Immun.

[36] Bekhbat M, Neigh GN (2018). Sex differences in the neuro-immune consequences of stress: focus on depression and anxiety. Brain Behav Immun.

[37] Miller GE, Cohen S, Pressman S (2004). Psychological stress and antibody response to influenza vaccination: when is the critical period for stress, and how does it get inside the body?. Psychosom Med.

[38] Potter JM, O’Donnell B, Carman WF (1999). Serological response to influenza vaccination and nutritional and functional status of patients in geriatric medical long-term care. Age Ageing.

[39] Remarque EJ, Cools HJM, Boere TJ (1996). Functional disability and antibody response to influenza vaccine in elderly patients in a Dutch nursing home. BMJ.

[40] Cunha LL, Perazzio SF, Azzi J (2020). Remodeling of the immune response with aging: immunosenescence and its potential impact on COVID-19 immune response. Front Immunol.

[41] Ciabattini A, Nardini C, Santoro F (2018). Vaccination in the elderly: the challenge of immune changes with aging. Semin Immunol.

[42] Franceschi C, Campisi J (2014). Chronic inflammation (inflammaging) and its potential contribution to age-associated diseases. J Gerontol Ser A Biol Sci Med Sci.

[43] Santoro A, Martucci M, Conte M (2020). Inflammaging, hormesis and the rationale for anti-aging strategies. Ageing Res Rev.

[44] Márquez EJ, Chung CH, Marches R (2020). Sexual-dimorphism in human immune system aging. Nat Commun.

[45] Milan-Mattos JC, Anibal FF, Perseguini NM (2019). Effects of natural aging and gender on pro-inflammatory markers. Braz J Med Biol Res = Rev Bras Pesqui medicas e Biol.

[46] Watson S, Caster O, Rochon PA, den Ruijter H (2019). Reported adverse drug reactions in women and men: aggregated evidence from globally collected individual case reports during half a century. EClinicalMedicine.

