# Supplementary material for: Sex differences in the efficacy and safety of SARS-CoV-2 vaccination in residents of long-term care facilities: insights from the GeroCovid Vax study
Source: Intern Emerg Med. 2023 Apr 29;18(5):1337–47. doi: 10.1007/s11739-023-03283-y (PMC10148701; doi:10.1007/s11739-023-03283-y)
Supplement: Supplementary file 1 — (DOCX 31 KB) [file 11739_2023_3283_MOESM1_ESM.docx]

**SUPPLEMENTARY MATERIAL**

**Online resource 1. Complete list of the GeroCovid Vax working group members (in alphabetical order)**

Angela Marie Abbatecola, Domenico Andrieri, Raffaele Antonelli Incalzi, Francesca Arenare, Viviana Bagalà, Tatjana Baldovin, Riccardo Bernardi, Alessandra Bianchi, Paola Bianchi, Raffaella Bisceglia, Ivan Bissoli, Fabio Bontempi, Gilda Borselli, Luigi Bottaro, Elisa Bottoni, Silvia Brandi, Claudio Bravin, Maria Adele Buizza, Carmine Cafariello, Alessia Maria Calabrese, Valeria Calsolaro, Marta Canepa, Carla Capasso, Mariagrazia Capuano, Sebastiano Capurso, Gabriele Carbone, Marialudovica Carducci, Silvia Carino, Nicoletta Cattaneo, Francesco Ceravolo, Maria Angelica Dorotea Chiesara, Danila Clerici, Pierpaolo Clerici, Alessandra Coin, Vieri Collacchioni, Mauro Colombo, Michela Compiano, Giuseppina Costanza, Giovanna Crupi, Roberta Cucunato, Manuela Marina D'Abramo, Emilia D'Agostino, Ferdinando D'Amico, Antonio De Simone, Stefania Del Vecchio*,* Maria Deleo, Annalaura Dell'Armi, Tommasina Di Brango, Anna Di Lonardo, Maria Raffaella Di Nanno, Babette Dijk, Luisa Elmo, Giorgio Fedele, Marisa Ferraro, Christian Ferro, Claudia Fiorucci, Francesca Fortunato, Pasquale Froncillo, Domenico Galasso, Nicola Galdiero, Caterina Galdiero, Stefania Gallo, Pier Paolo Gasbarri, Maria Grazia Gennai, Giuliana Ghiselli Ricci, Elisa Giribaldi, Carmen Godeanu, Samuele Gommaraschi, Roberta Granata, Giada Ida Greco, Angela Greco, Antonio Grillo, Gianbattista Guerrini, Mauro Guglielmo, Labjona Haxhiaj, Claudio Giuseppe Iacovella, Marina Indino, Valerio Alex Ippolito, David Kanah, Liudmila Kountsevich, Jovan Leci, Federica Limongi, Agata Lipari, Vincenzo Longo, Stefania Maggi, Alba Malara, Leonarda Maltese, Maria Marotta, Giuseppe Mazzarella, Hior Melnik, Pasquale Minchella, Paolo Moneti, Fabio Monzani, Walter Morandotti, Francesco Morelli, Maria Grazia Mortola, Marianna Noale, Chukwuma Okoye, Graziano Onder, Patrizia Orlanducci, Barbara Paganelli, Michele Pagano, Nicola Pagano, Raffaele Palladino, Annapina Palmieri, Magda Palumeri, Simone Paolini, Raimondo Paternò, Angela Pavan, Loris Pelucchi, Agostino Perri, Francesco Perticone, Rosanna Pesce, Sabrina Pigozzo, Francesco Pili, Rosa Prato, Rosanna Pullia, Ahmad Amedeo Qasem, Francesco Raffaele Addamo, Cecilia Raffaelli, Vincenzo Restivo, Michela Fernanda Rigon, Franco Romagnoni, Carmine Romaniello, Valentina Romano, Maria Cristina Ruberto, Marcello Russo, Bruno Sala, Sara Sambo, Maria Concetta Sciurti, Antonietta Scriva, Luca Secchi, Vincenzo Settembrini, Federica Sirianni, Deborah Spaccaferro, Fausto Spadea, Manuela Stefanelli, Paola Stefanelli, Brunella Stelitano, Stefania Stringhini, Andrea Tarsitano, Camilla Terziotti, Caterina Trevisan, Rita Ursino, Giovanni Veneziano, Maria Teresa Vigliotta, Marco Vignati, Eva Vignola, Enrico Virgilio, Maria Visconti, Stefano Volpato, Susanna Vozzi, Sabrina Zaccone.

**Online resource 2. Frequency of adverse events in the first 7 days after the 1^st^, 2^nd,^ and booster vaccine dose in male and female participants**

|  | **Adverse events after the 1^st^ vaccine dose** | | | | **Adverse events after the 2^nd^ vaccine dose** | | | | **Adverse events after the booster dose** | | | |
| --- | --- | --- | --- | --- | --- | --- | --- | --- | --- | --- | --- | --- |
|  | **Males** | **Females** | **p** | **Males** | | **Females** | **p** | **Males** | | **Females** | **p** |  |
| n | 920 | 2280 |  | 745 | | 1797 |  | 552 | | 1504 |  |  |
| Fever | 23 (2.5) | 62 (2.7) | 0.82 | 18 (2.4) | | 46 (2.6) | 0.943 | 13 (2.4) | | 30 (2.0) | 0.74 |  |
| Muscle weakness | 20 (2.2) | 38 (1.7) | 0.408 | 8 (1.1) | | 19 (1.1) | 1 | 7 (1.3) | | 13 (0.9) | 0.567 |  |
| Low-grade fever | 19 (2.1) | 35 (1.5) | 0.367 | 11 (1.5) | | 22 (1.2) | 0.75 | - | | - | - |  |
| Muscles and joints pain | 36 (3.9) | 86 (3.8) | 0.931 | 20 (2.7) | | 45 (2.5) | 0.901 | 9 (1.6) | | 26 (1.7) | 1 |  |
| Pain and swelling at the injection site | 70 (7.6) | 219 (9.6) | 0.086 | 44 (5.9) | | 129 (7.2) | 0.283 | 18 (3.3) | | 43 (2.9) | 0.742 |  |
| Site injection itching | 18 (2.0) | 62 (2.7) | 0.26 | 10 (1.3) | | 29 (1.6) | 0.742 | 3 (0.5) | | 18 (1.2) | 0.29 |  |
| Headache | 6 (0.7) | 25 (1.1) | 0.336 | 6 (0.8) | | 20 (1.1) | 0.628 | 4 (0.7) | | 9 (0.6) | 0.995 |  |
| Swollen lymph nodes | 0 (0.0) | 7 (0.3) | 0.206 | 0 (0.0) | | 0 (0.0) | - | 2 (0.4) | | 5 (0.3) | 1 |  |
| Chills | 10 (1.1) | 6 (0.3) | 0.007 | 3 (0.4) | | 9 (0.5) | 0.991 | 0 (0.0) | | 3 (0.2) | 0.69 |  |
| Site injection redness | 26 (2.8) | 105 (4.6) | 0.028 | 12 (1.6) | | 53 (2.9) | 0.071 | 1 (0.2) | | 5 (0.3) | 0.919 |  |
| Difficulty breathing | 3 (0.3) | 15 (0.7) | 0.382 | 2 (0.3) | | 4 (0.2) | 1 | 1 (0.2) | | 6 (0.4) | 0.746 |  |
| Insomnia | 14 (1.5) | 27 (1.2) | 0.552 | 11 (1.5) | | 17 (0.9) | 0.338 | 5 (0.9) | | 15 (1.0) | 1 |  |
| Sneezing | 2 (0.2) | 2 (0.1) | 0.699 | 0 (0.0) | | 1 (0.1) | 1 | 2 (0.4) | | 2 (0.1) | 0.63 |  |
| Fast Heart Rate | 0 (0.0) | 3 (0.1) | 0.644 | 0 (0.0) | | 6 (0.3) | 0.258 | 0 (0.0) | | 2 (0.1) | 0.953 |  |
| Cough | 5 (0.5) | 11 (0.5) | 1 | 6 (0.8) | | 2 (0.1) | 0.014 | 4 (0.7) | | 7 (0.5) | 0.709 |  |
| Anorexia | 7 (0.8) | 21 (0.9) | 0.818 | 6 (0.8) | | 17 (0.9) | 0.912 | 5 (0.9) | | 4 (0.3) | 0.116 |  |
| Nausea or vomiting | 4 (0.4) | 17 (0.7) | 0.457 | 5 (0.7) | | 20 (1.1) | 0.42 | 1 (0.2) | | 11 (0.7) | 0.261 |  |
| Delirium | 9 (1.0) | 35 (1.5) | 0.291 | 4 (0.5) | | 12 (0.7) | 0.917 | 4 (0.7) | | 18 (1.2) | 0.496 |  |
| Diarrhea | 9 (1.0) | 41 (1.8) | 0.125 | 4 (0.5) | | 21 (1.2) | 0.212 | 4 (0.7) | | 19 (1.3) | 0.428 |  |
| Increased BP | 2 (0.2) | 8 (0.4) | 0.793 | 3 (0.4) | | 3 (0.2) | 0.505 | 0 (0.0) | | 6 (0.4) | 0.305 |  |
| Weakness | 28 (3.0) | 53 (2.3) | 0.295 | 18 (2.4) | | 43 (2.4) | 1 | 10 (1.8) | | 25 (1.7) | 0.968 |  |
| Cutaneous rash | 1 (0.1) | 5 (0.2) | 0.839 | 1 (0.1) | | 2 (0.1) | 1 | 0 (0.0) | | 4 (0.3) | 0.517 |  |
| Confusion | 12 (1.3) | 26 (1.1) | 0.836 | 5 (0.7) | | 12 (0.7) | 1 | 1 (0.2) | | 6 (0.4) | 0.746 |  |
| Dizziness | 1 (0.1) | 6 (0.3) | 0.668 | 4 (0.5) | | 5 (0.3) | 0.527 | 0 (0.0) | | 1 (0.1) | 1 |  |

*Abbreviations:* BP, blood pressure. *Notes*. P-values refer to the comparison between males and females.

**Online resource 3. Incident SARS-CoV-2 infections and their severity in male and female residents over the 12-month follow-up**

|  | **Males**  **(n=941)** | **Females**  **(n=2318)** |
| --- | --- | --- |
| Uninfected | 818 (86.9) | 2046 (88.3) |
| SARS-CoV-2 infection | 123 | 272 |
| *Asymptomatic* | 109 (11.6) | 239 (10.3) |
| *Mild disease with no O2-therapy needs* | 12 (1.3) | 32 (1.4) |
| *Mild or severe disease with O2-therapy needs* | 0 (0) | 0 (0) |
| *Death* | 2 (0.2) | 1 (0.04) |

*Notes*. Numbers are counts (%). At the Chi-squared test, no significant differences between men and women emerged in the incidence (p=0.311) and severity (p=0.354) of SARS-CoV-2 infections.

|  | **Hazard ratio (95% confidence interval)**  **p-value** | |
| --- | --- | --- |
|  | **Model 1** | **Model 2** |
| Sex (female vs male) | 1.01 (0.80, 1.24)  p=0.960 | 1.08 (0.73, 1.18)  p=0.550 |

**Online resource 4. Cox regression for the association between sex and incident SARS-CoV-2 infection over the 12-month follow-up**

Model 1 is adjusted for age. Model 2 is also adjusted for ethnic origin, mobility level, previous SARS-CoV-2 infection, number of vaccine doses received, cognitive disorders, and number of chronic diseases.

**Online resource 5. Baseline characteristics of the male and female study participants included in the subsample serological monitoring**

|  | | | **Males**  **(n=160)** | | | **Females**  **(n=364)** | **p-value** |
| --- | --- | --- | --- | --- | --- | --- | --- |
| Age (years) | | | 76.30 (9.81) | | | 85.02 (8.40) | <0.001 |
| Ethnic origin (Caucasic) | | | 154 (96.2) | | | 357 (98.1) | 0.447 |
| Mobility level |  |  | |  |  | | 0.044 |
| Walks independently or with aids | | | 97 (60.6) | | | 178 (48.9) |  |
| Moves with a wheelchair or bedridden | | | 46 (28.7) | | | 140 (38.5) |  |
| *Chronic diseases* | | |  | | |  |  |
| Hypertension | | | 94 (58.8) | | | 252 (69.2) | 0.026 |
| Cardiovascular diseases | | | 60 (37.5) | | | 184 (50.5) | 0.008 |
| Peripheral artery disease | | | 17 (10.6) | | | 41 (11.3) | 0.949 |
| Cerebrovascular diseases | | | 22 (15.3) | | | 45 (13.9) | 0.81 |
| Chronic respiratory diseases | | | 39 (24.4) | | | 57 (15.7) | 0.024 |
| Diabetes mellitus | | | 38 (23.8) | | | 82 (22.5) | 0.846 |
| Poor Nutritional Status | | | 26 (17.7) | | | 39 (12.0) | 0.126 |
| Obesity | | | 8 (5.0) | | | 33 (9.1) | 0.156 |
| Chronic Liver Disease | | | 7 (4.8) | | | 18 (5.5) | 0.905 |
| Immune System Disorder | | | 6 (4.1) | | | 7 (2.1) | 0.375 |
| Inflammatory Bowel Disease | | | 3 (2.0) | | | 8 (2.5) | 1 |
| Cancer | | | 11 (7.5) | | | 26 (8.0) | 1 |
| Osteoarticular diseases | | | 45 (28.1) | | | 191 (52.5) | <0.001 |
| Chronic Renal Failure | | | 20 (13.6) | | | 38 (11.7) | 0.655 |
| Urologic diseases | | | 36 (22.5) | | | 1 (0.3) | <0.001 |
| Cognitive disorders | | | 92 (57.5) | | | 236 (64.8) | 0.134 |
| Parkinson's disease or parkinsonism | | | 20 (12.5) | | | 21 (5.8) | 0.014 |
| Depressive disorders | | | 58 (36.2) | | | 161 (44.2) | 0.107 |
| Anxiety disorders | | | 38 (23.8) | | | 67 (18.4) | 0.197 |
| N. chronic diseases | | | 4.66 (2.41) | | | 4.97 (2.29) | 0.181 |
| *SARS-CoV-2 vaccine type (first doses)* | | |  | | |  | 0.124 |
| Moderna | | | 14 (8.8) | | | 51 (14.0) |  |
| Comirnaty | | | 146 (91.2) | | | 313 (86.0) |  |
| *Number of vaccine doses received* | | |  | | |  | 0.047 |
| 1 | | | 10 (6.2) | | | 23 (6.3) |  |
| 2 | | | 81 (50.6) | | | 143 (39.3) |  |
| 3 | | | 69 (43.1) | | | 198 (54.4) |  |
| Previous COVID-19 | | | 65 (42.2) | | | 132 (37.8) | 0.407 |
